# Fetal blockade of nicotinic acetylcholine transmission causes autism-like impairment of biological motion preference in the neonatal chick

**DOI:** 10.1093/texcom/tgac041

**Published:** 2022-11-18

**Authors:** Toshiya Matsushima, Momoko Miura, Nina Patzke, Noriyuki Toji, Kazuhiro Wada, Yukiko Ogura, Koichi J Homma, Paola Sgadò, Giorgio Vallortigara

**Affiliations:** Department of Biology, Faculty of Science, Hokkaido University, Sapporo 060-0810, Japan; Faculty of Pharmaceutical Science, Health Science University of Hokkaido, Tobetsu 061-0293, Japan; Center for Mind/Brain Sciences, University of Trento, Rovereto 38068, Italy; Department of Biology, Faculty of Science, Hokkaido University, Sapporo 060-0810, Japan; Faculty of Pharmaceutical Science, Health Science University of Hokkaido, Tobetsu 061-0293, Japan; Department of Biology, Faculty of Science, Hokkaido University, Sapporo 060-0810, Japan; Health and Medical University, Potsdam 14471, Germany; Department of Biology, Faculty of Science, Hokkaido University, Sapporo 060-0810, Japan; Department of Biology, Faculty of Science, Hokkaido University, Sapporo 060-0810, Japan; Graduate School of Information Science and Technology, The University of Tokyo, Tokyo 113-8654, Japan; Japan Science and Technology Agency, PRESTO, Kawaguchi 332-0012, Japan; Department of Molecular Biology, Faculty of Pharmaceutical Sciences, Teikyo University, Tokyo 173-8605, Japan; Center for Mind/Brain Sciences, University of Trento, Rovereto 38068, Italy; Center for Mind/Brain Sciences, University of Trento, Rovereto 38068, Italy

**Keywords:** autism spectrum disorder, biological motion, imprinting, neonicotinoid, valproic acid

## Abstract

Several environmental chemicals are suspected risk factors for autism spectrum disorder (ASD), including valproic acid (VPA) and pesticides acting on nicotinic acetylcholine receptors (nAChRs), if administered during pregnancy. However, their target processes in fetal neuro-development are unknown. We report that the injection of VPA into the fetus impaired imprinting to an artificial object in neonatal chicks, while a predisposed preference for biological motion (BM) remained intact. Blockade of nAChRs acted oppositely, sparing imprinting and impairing BM preference. Beside ketamine and tubocurarine, significant effects of imidacloprid (a neonicotinoid insecticide) appeared at a dose ≤1 ppm. In accord with the behavioral dissociations, VPA enhanced histone acetylation in the primary cell culture of fetal telencephalon, whereas ketamine did not. VPA reduced the brain weight and the ratio of NeuN-positive cells (matured neurons) in the telencephalon of hatchlings, whereas ketamine/tubocurarine did not. Despite the distinct underlying mechanisms, both VPA and nAChR blockade similarly impaired imprinting to biological image composed of point-light animations. Furthermore, both impairments were abolished by postnatal bumetanide treatment, suggesting a common pathology underlying the social attachment malformation. Neurotransmission via nAChR is thus critical for the early social bond formation, which is hindered by ambient neonicotinoids through impaired visual predispositions for animate objects.

## Introduction

Despite heterogeneous diagnostic phenotypes, autism spectrum disorder (ASD) is primarily characterized by impaired social interactions (Wing and Gould 1979; DSM-52013). A visual predisposition expressed by a preference for animate objects (such as attraction for face-like and biological motion [BM] displays) typically arises early in life (Simion et al. 2008; Bardi et al. 2011; Bidet-Ildei et al. 2014; Sifre et al. 2018; Di Giorgio et al. 2021), which is hampered in neonates/juveniles with ASD or its familial risk (Rutherford et al. 2006; Klin et al. 2009; Pavlova 2012; Di Giorgio et al. 2016; Wang et al. 2018). Along with genetic factors, exposure to environmental chemical agents, such as the anticonvulsant valproic acid (VPA) (Moore et al. 2000; Rasalam et al. 2005; Bromley et al. 2013; Christensen et al. 2013) and pesticides (Keil et al. 2014; Gunier et al. 2017; von Ehrenstein et al. 2019; Ongono et al. 2020) during pregnancy is an ASD risk factor. Despite intensive efforts to develop mammalian models to assess these environmental factors (Nicolini and Fahnestock 2018; Chaliha et al. 2020), issues remain to be solved because these neonatal mammals (mostly rodents) do not spontaneously exhibit preferences for animate stimuli, such as BM in early life, which is found in human neonates (Simion et al. 2008) and a taxonomically distant animal, newly hatched domestic chicks (Vallortigara et al. 2005). Various nonhuman animals discriminate BM point-light animations (Dittrich et al. 1998; Parron et al. 2007; MacKinnon et al. 2010; Nunes et al. 2020), but their BM perception does not spontaneously arise after birth.

In chicks, the BM preference is enhanced by imprinting to an artificial (non-BM) object (Miura and Matsushima 2012), thereafter facilitates and canalizes subsequent imprinting (Miura and Matsushima 2016; Miura et al. 2020). Along with the commonality in the early development of BM, humans (Troje and Westhoff 2006) and chicks (Vallortigara and Regolin 2006) show inversion effects, namely predisposition to up-right walking configuration over the inversed (upside down) display. Even taking into account the different brain organizations between birds and humans, which, however, seems to be less than it appeared to be (Güntürkün and Bugnyar 2016), the functional and developmental similarities are striking. Although the neural substrates of the neonatal BM perception are largely unknown, recent studies implicate subcortical visual processing in evolutionarily conserved visual systems (Chang et al. 2018; Hirai and Senju 2020). Preattentive processing of biologically relevant stimuli (such as BM and face configuration) could canalize the subsequent memorization, leading to the adaptive formation of social attachment to specific individuals.

What prenatal processes construct the BM preference? Previous studies have revealed effects of fetal VPA application on social behaviors in chicks (Nishigori et al. 2013; Lorenzi et al. 2019; Sgadò et al. 2018; Adiletta et al. 2021), but the effect on BM preference has not been investigated. Furthermore, if the effect of VPA on the development of social behavior was mediated by well-documented action as an inhibitor of histone deacetylation (Phiel et al. 2001), what behavioral effects might other risk agents have? As VPA is a potent anticonvulsant drug, it might effectively suppress fetal movements and thereby the BM preference. As a first step, we hypothesized that suppression of spontaneous fetal movements (Bekoff et al. 1975; Blankenship and Feller 2010) is responsible, where excitatory gamma-aminobutyric acid (GABA) actions could play a role (Ben-Ari 2002; Represa and Ben-Ari 2005). In human adults, execution of unfamiliar motor patterns facilitates the perception of the corresponding BM animations, thus, nonvisual motor execution might be involved (Casile and Giese 2006). According to the motor involvement, an imaging study suggested the contribution of the posterior part of cerebellum in BM perception (Jack et al. 2017). If this applies also to fetuses, chemical agents that suppressed spontaneous movements, including the blockade of neuro-muscular transmission, may impair the development of BM preference. We started this study by testing chemical agents that effectively suppress the fetal movement at embryonic day 14 (E14), the stage wherein VPA is reported to be effective (Nishigori et al. 2013).

## Materials and methods

### Animals

Domestic white Leghorn chicks (*Gallus gallus domesticus*, an egg-laying strain of White Leghorn locally referred to as “Julia”) were used. Fertilized eggs were purchased from a hatchery (Iwamura Co., Niigata/Hokkaido Japan, 20–40 eggs per batch at a time) every 2 weeks, and the batch was numbered. Eggs were incubated in the laboratory by using type P-008B incubators (Showa Furanki Co., Saitama Japan) with its temperature controlled at 37.7 °C and the humidity maintained at ca. 80%. Incubation started within 2 weeks of the arrival of eggs. The inside of the incubator was kept in complete darkness until hatch. To avoid posthatch visual experiences, hatchlings were individually housed in black plastic boxes placed in another incubator of the same type, which were kept in darkness until the experiment at 18–36 h after hatch. Chicks were sexed after the experiment. Generally, fertilized eggs weighed 59.8 ± 3.1 g (mean ± s.d., *n* = 170) and ca. 2 g lighter at E14, whereas the shells weighed 8.0 ± 0.1 g (*n* = 16), so the wet weight is assumed to be around 50 g. In this series of experiments, as the egg weight did not considerably vary, the amount of injected chemical per egg was fixed and was not adjusted for each egg.

Eggs that failed to show normal development or active fetal movements were discarded on E14. Those chicks that failed to run more than 20 times (i.e. 30 cm × 20 = 600 cm) during the training periods (2 h in total) were discarded and not tested. Those chicks that run at training >20 but did not move at all during all test sessions (~2% of the successfully trained chicks) were discarded post hoc after the test. Total number of eggs and chicks used will be indicated separately below in each section. Individual chicks were coded, and the behavioral experiments were accomplished by experimenter blind to the treatment (see Supplementary Material for detail).

### Compliance with ethical standard

Experiments were conducted under the guidelines and approval of the Committee on Animal Experiments of Hokkaido University (approval number 20-0141). The guidelines are based on the national regulations for animal welfare in Japan (Law for Humane Treatment and Management of Animals after partial amendment No. 68, 2005). All subjects were sexed, and the number of subjects used are indicated in each experiment.

### Ballistographic recording of fetal movements (experiment 1)

**Fig. 1:**
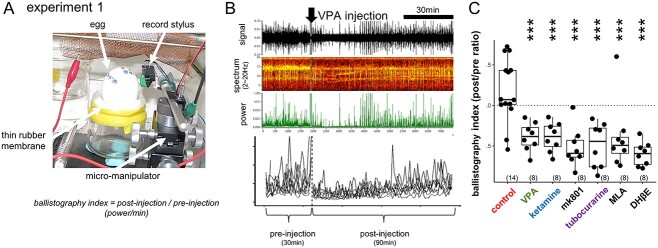
Suppression of the spontaneous fetal movement by VPA and other chemicals. A, B) Ballistography recording (experiment 1) and blockade by sodium valproate (VPA). Record stylus placed against the eggshell surface detected the miniature vibration caused by spontaneous fetal movement. C) The dose was experimentally searched for each agent so that fetal movement was similarly suppressed on E14, wherein the treatment gave rise to normal hatch rate and successful training of the hatchlings. Asterisks indicate the significant difference from the control by multiple regression analysis; ^*^, *P* < 0.05, ^*^^*^, *P* < 0.01, ^*^^*^^*^, *P* < 0.001 in this and the following figures. Number in parenthesis indicates the sample size.

Mechanical vibration caused by spontaneous fetal movements were recorded following the ballistocardiogram method developed by Suzuki et al. (1989). By using a micromanipulator, an analog record stylus cartridge (type AT VM95E, Audio Technica Co., Tokyo Japan) was placed against the shell of egg settled on a thin rubber membrane, which was stretched on a glass jar (Fig. 1A). The jar and the manipulator were fixed on an iron plate and were housed in an incubator controlled at 37.7 °C and high humidity. The recorded monoaural signal was amplified by a hand-made low input-impedance amplifier (made of FET operational amplifier, TL084), band-pass-filtered (cut-off frequency: 10-100 Hz at 8 dB per octave, gain: x1,000), and stored at 1,000 point/s sampling rate by Spike2 (ver.7, the interface CED-1401 micro3, Cambridge Electric Design Co., Cambridge UK). Frequency spectrum of 2–20 Hz range was monitored, and the converted power was stored every 1 s (Fig. 1B). Using R as platform (see below), the power value was averaged every 1 min, and the temporal profiles were constructed for preinjection 30 min and postinjection 90 min for each egg, thus yielding the ratio of pre-/postinjection power/min as ballistography index (Fig. 1C).

Superimposed traces of the ballistography obtained from 9 groups of fetuses are shown in Supplementary Fig. S1A. In addition, dose dependency of the suppressive effects of VPA, tubocurarine, and IMI was examined (Supplementary Fig. S1B). Sample size was arbitrarily set as *n* = 8 for each group of chemical/dosage, and the present study is based on the recordings obtained from 110 eggs in total.

### Apparatus for imprinting and tests (experiments 2 and 3)

An I-shaped maze (10 cm wide, 70 cm long) was equipped with a 50-cm long treadmill consisting of a rubber belt at the center and an LCD monitor at each end and placed in a dark room at 27~28 °C. See our previous reports for details (Miura et al. 2018, 2020; Takemura et al. 2018).

Two training sessions (1 h each) were given at an 1-h interval. An infrared sensor and a transparent Plexiglass partition were placed at a point 10 cm from 1 monitor, and the other monitor was occluded by an opaque partition. A loudspeaker behind the LCD monitor emitted mechanical sound synchronized with the toy movement on the screen. When chicks approached the monitor and hit the sensor, the rubber belt of the treadmill moved for a short period of 1.0 s, drawing the chick backward by about 30 cm at a time with the shortest intervals set at 0.1~2 s. The apparatus was controlled by Arduino. The treadmill motions were digitally counted, and the number of approaches was recorded for each chick. We monitored the chick behavior via a video camera set at the ceiling.

In the machine used by Miura M, the interval of treadmill was 0.1, whereas it was 2 s in the machine Matsushima T; the different setting was not intentional but was done by mistake. Post hoc analysis revealed the difference in the total run numbers during training, but there was no difference in the test scores; data were therefore merged.

After 30-min to 1-h pause period in a dark chamber kept at ~ 30 °C, the trained chicks were tested in binary choice using the same apparatus without treadmill motions. In the BM test, 1 monitor displayed a linear point-light animation and the other monitor a walking one, both composed of white lights. In the subsequent imprinting test, 1 monitor displayed red toy (familiar object) and another yellow (novel object), both unaccompanied by the mechanical sound used in training. The chick was supposed to choose 1 side when the whole body was located close (<30 cm) to the monitor (delineated by dashed line). Five-minute tests were repeated twice after swapping the side, and the initial side was counterbalanced among individuals. Tests were video-recorded. Intervals between tests were set at 90–120 s. The difference in stay times gave the choice score in seconds, thus ranging from −600 to +600 s.

**Fig. 2:**
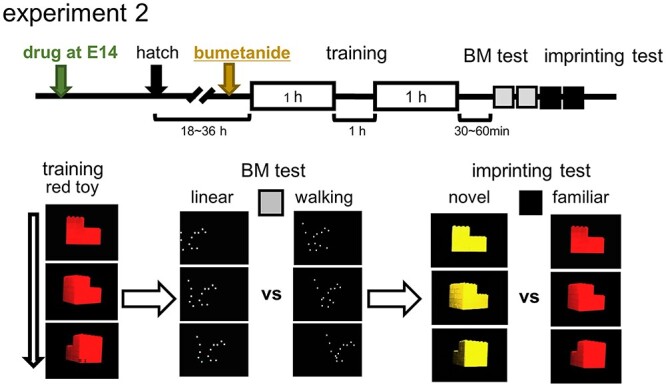
BM preference and imprinting; training and test procedures (experiment 2). Chicks were trained by an artifact object (rotating red toy) and tested by binary choices for BM (walking motion vs. linear motion) and imprinting (red toy vs. yellow); stay time difference (s) was used for the scores.

In experiment 2, chicks were trained and tested on posthatch 1 (P1) day (at 18~36 h after hatch) using the procedure shown in Fig. 2. In experiment 3, chicks were similarly trained and tested for the BM preference on P1, then re-trained on P2 (24 h later) by merged Lp[red] and Wp[yellow] (Fig. 5); the chicks were simultaneously exposed to 2 point-light animations, 1 walking in yellow, Wp[yellow], and another linear in red, Lp[red]. Our study (Miura et al. 2020) revealed that chicks exposed to the merged animations showed significantly higher preference to Wp[yellow] over Lp[red], if the BM preference of the chicks had been induced by the training using the red toy on P1.

For behavioral experiments, group size (number of chicks) was set at 10 for each chemical/dosage applied based on a pilot series of experiment (see Supplementary Material for details). Behavioral experiments were not replicated but performed once for each of the chemicals/doses examined. The IMI treatment (E0) was replicated twice, 12 eggs for each dosage, and all the data were merged.

### Video clips and point-light animations for training and tests

Two video clips of a rotating toy and 3 point-light animations were prepared as in our previous reports (Miura and Matsushima 2016; Miura et al. 2020); see below for the list. Video clips were displayed on black background at a speed of 30 frames/s. We made editing by Adobe Premiere (elements 7) and the color was set either to red (R: 255, G: 0, B: 0) or yellow (R: 255, G: 255, B: 0). These stimuli were displayed on the LCD monitors (size 10.4″, 800 pixels × 600 pixels, Logitec LCM-T102AS, Japan; flash rate: 56–75 Hz, brightness: 230 cd/m2, pitch size: 0.264 mm × 0.264 mm) using free viewer software (A-player, version 6.0) on PC. The width of the presentation was set at 10.5 cm on the monitor, the surface of which was placed at 2.0 cm away from the terminal window of the maze. See Supplementary Material for the list.

### Chemical agents

Eggs (E14, if not stated otherwise) received single injection of 200 μL solution to the air chamber through 1 of 2 holes on the round edge of the shell; another hole served as an air vent for smooth injection. The holes were sealed by mending tape. For each agent, the highest dosage was determined, which (i) did not reduce the rate of hatching and (ii) significantly suppressed the spontaneous fetal motions, and then the 1/5 diluted solution was tested. Control eggs received injection of the same amount of vehicle, distilled water. See Supplementary Material for the list.

### Histone acetylation assay in cultured brain cells (experiment 4)

Embryos (E7 and E14) were aseptically harvested from eggs and brain tissues were separated. Brain tissues were finely minced with scissors in sterile phosphate-buffered saline (PBS) and dissociated by pipetting with micropipette tips. Dissociated brain tissues were suspended in a brain culture medium composed of DMEM/F12 (048-29785, FUJIFILM Wako Pure Chemical Co., Tokyo, Japan), 10% fetal bovine serum (S-FBS-NL-015, Serana Europe GmbH, Brandenburg, Germany), 1× Antibiotic-Antimycotic (15240–096, Life Technologies Co., CA, United States) and were maintained on coverslip glasses coated with collagen type I-C (631-00771, FUJIFILM Wako Pure Chemical Co., Tokyo, Japan) for 4 days at 37 °C in 5% CO_2_ condition.

The cultured brain tissues were incubated with ketamine (66 or 6.6 μg/mL), VPA (1.2 mM or 0.12 mM) or vehicle PBS in a brain culture medium without FBS for 2 h at 37 °C in 5% CO_2_. After incubation, cultured brain tissues were fixed with 4% paraformaldehyde in PBS for 5 mins, blocked with a blocking solution (4% normal goat serum (S-1000, VECTOR laboratories Inc., CA, United States), 1% blocking reagent (1096176, Roche Ltd, Basel, Swiss), and 0.5% Tween20 in PBS for 30 min and then incubated with 1:3,000 diluted Antiacetyl Histone H3 (Lys27), mouse monoclonal antibody (MA309A, TaKaRa Bio Inc., Shiga, Japan) in the blocking solution for 16 h at 4 °C in a humidity chamber. Following the first antibody incubation, tissues were incubated with 1:2,000 diluted Goat antimouse IgG, Alexa Fluor 555 antibody (A21422, ThermoFisher Scientific, MA, United States) in the blocking solution for 2 h at room temperature in a humidity chamber. Stained tissues were mounted in Mounting Medium with DAPI (H-1200, VECTOR Laboratories Inc.) and were imaged using a BZ-X810 microscope (Keyence Co., Osaka, Japan). In each duplicated experimental group, 3 fields of view were taken per coverslip, and the acetylation levels of H3K27 in the nuclei of 100 randomly selected cells from 6 fields of view were measured as fluorescence intensity using Image J. Experiment 4 was accomplished once and not replicated.

### Brain weight and isotropic fractionation measurement of brain cells (experiments 5 and 6)

The P1 chicks were transcardially perfused by 4% paraformaldehyde in 0.1 M of phosphate buffer under a deep anesthesia by i.m. injection of 0.8 mL of ketamine-xylazine cocktail; a 1:1 mixture of 10 mg/mL of ketamine hydrochloride (Daiichi-Sankyo Propharma Co.) and 2 mg/mL of xylazine (Sigma-Aldrich Co.).

The whole brain (cut at the caudal end of medulla oblongata) was dissected and postfixed in the fixative for overnight, stored for 2~4 days in PBS at 4 °C and weighed. The telencephalon was isolated at the rostral level of diencephalon and manually chopped in small pieces by scalpel. The tissue was weighed and homogenized in a detergent solution (40 mM sodium citrate and 1% Triton X-100) using a 7-mL glass Tenbroeck tissue homogenizer. The homogenate and several washes of the homogenizer were transferred to a 50-mL falcon tube using a glass pipette. To visualize cell nuclei, DAPI (4,6-diamidino-2-phenylindoledihydrochloride, Invitrogen, Carlsbad, Calif., United States) was added to the suspension from a stock solution at 20 mg/L (dilution 1:20–1:50), and the final volume of the suspension was recorded. To estimate the total amount of cells in the suspension, the nuclear density was quantified in a Neubauer chamber by a fluorescent microscope (BX-50, Olympus Co., Tokyo Japan).

To estimate the number of matured neuron, aliquot (500 μL) of the nuclear suspension washed with PB (0.1 M) and incubated overnight in the dark at 4 °C with Cy3-labeled rabbit polyclonal neuronal nuclear antigen antibody (1:150 dilution; NeuN, RRID:AB_11204707). The percentage of neuronal nuclei was determined by counting at least 500 DAPI-stained nuclei and establishing the fraction that was also NeuN-positive according to the protocol developed by Herculano-Houzel and Lent (2005). Briefly, the total number of neurons in each sample was determined by multiplying the total number of cells in the structure by the NeuN-positive fraction obtained. The total number of nonneuronal cells was determined by subtracting the total number of neurons from the total number of cells. Densities of neurons and nonneurons (cell/mg) were determined by dividing the number of neurons or nonneurons by the mass (mg) of the structure. Experiments 5 and 6 were accomplished once and were not replicated.

### Statistical analyses

Statistical computations were performed on RStudio version 3.6.3 (2020 February 29). For single and multiple regression analyses, linear models were constructed using the function “lm()” with the control group as the reference set; 1 model was calculated for each set of experimental groups. Multiple regression, including variables such as “sex,” “batch,” and “run” (locomotor counts during training), appeared not to improve the statistical judgments; we therefore adopted single regression by “drug” (and dose) in most cases. Outliers were not discarded in all of the experiments 1–6. Results of statistical computations are summarized in the Supplementary Material.

Significance is coded as 0 < ^*^^*^^*^ < 0.001 < ^*^^*^ < 0.01 < ^*^ < 0.05, and ns means *P*-value ≥0.05. The raw data set, the R codes for statistic computations and the video clips / point light animations used in this study are available at data repository site of the Hokkaido University (https://eprints.lib.hokudai.ac.jp/dspace/handle/2115/87070).

## Results

### Suppression of the spontaneous fetal movement by chemical agents

Fetal movement was reliably detected by placing an analog record stylus against the shell surface (experiment 1, Fig. 1A). Low frequency power (2~20 Hz) of the recorded signal (ballistogram) revealed that VPA injection to the air sac acutely suppressed the movement (Fig. 1B). Similar suppression was found by ketamine (sedative drug of a wide action spectrum including blockade of NMDAR and nicotinic acetylcholine receptor [nAChR]), mk801 (selective NMDAR blocker), and tubocurarine (nAChR blocker of a wide spectrum) (Fig. 1C; also see Supplementary Fig. S1A and B). Among other nAChR blockers, methyllycaconitine citrate (MLA, specific to neuronal α7 subtype), and dihydro-β-erythroidine hydrobromide (DHβE, specific to muscular α4β2 subtype) were similarly effective. Fetal movement is sensitive to the blockade of cholinergic or NMDAR-mediated neurotransmission in the brain (VPA, ketamine, mk801, and MLA) and neuro-muscular junctions in the periphery (DHβE).

### BM preference and imprinting were doubly dissociated in chicks

Hatchlings of the injected eggs were tested for BM preference and imprinting (experiment 2); see Supplementary Material for the determination of the sample size. Chicks were individually trained by a video clip of a rotating red toy (displayed on an LCD with sound) for 2 h with 1-h intermission. After 30–60 min, the trained chicks were tested for BM preference (walking point-light animation over linear motion, both in white) and for imprinting memory (familiar red toy over novel yellow; Fig. 2).

**Fig. 3:**
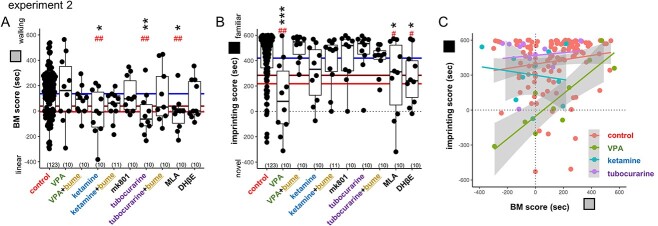
BM preference and imprinting; VPA and nAChR blockers (experiment 2). A) BM and B) imprinting score of the treated chicks are shown in box-plot of the median and quadrants with superimposed individual data. Horizontal colored lines indicate the critical levels of the control data determined by bootstrap computation; average (blue), 95% (brown), and 99% (red) confidence levels; see the Supplementary Fig. S4 for details. C) Imprinting scores were plotted against BM scores. Distinct spectrums were found between VPA and other agents. Asterisks (^*^, ^*^^*^, ^*^^*^^*^) indicate significance levels by multiple regression analysis. Sharp marks (#, ##) indicate significance levels based on the bootstrapping analysis.

Among the examined chemical agents, ketamine, tubocurarine, and MLA significantly reduced the BM preference compared with control in a dose-dependent manner, while VPA failed (Fig. 3A; Supplementary Fig. S4 for comparisons with the bootstrapping of the control data). Despite effective suppression of the fetal movement, mk801 failed to cause any behavioral effects, and hence, VPA and ketamine evidently acted through pathways other than NMDAR. Moreover, nAChRs are responsible for the BM preference impairment because ketamine, tubocurarine, and MLA were similarly effective; α7 receptor is a plausible candidate rather than α4ßn subtypes. Notably, besides NMDAR suppression, ketamine directly blocks nAChR-associated channels in the central nervous system and peripheral tissues (Scheller et al. 1996; McMillan and Muthukumaraswamy 2020). However, VPA spared BM and impaired imprinting (Fig. 3B), indicating that the underlying pharmacological mechanisms are doubly dissociated between these 2 behavioral traits except that MLA and DHβE weakly, but significantly, suppressed imprinting. Suppression of fetal movements is therefore not sufficient for the BM preference impairment in hatchlings.

Distinct behavioral phenotypes appeared when the imprinting score was plotted against the BM score (Fig. 3C). The control chicks showed a weak but significant positive correlation between the 2 scores (*r* = +0.21, *P* = 0.0217 for *n* = 123, Spearman’s rank correlation), which was stronger in the VPA chicks (*r* = +0.88, *P* = 0.000747 for *n* = 10) but absent in the ketamine (*r* = −0.042, *P* = 0.919) and the tubocurarine chicks (*r* = −0.10, *P* = 0.785). Although the cause of the individual variations is unknown, those chicks with high BM score were resistant to VPA applied on E14. On the other hand, ketamine and tubocurarine spared the imprinting score in these chicks with low BM scores.

**Fig. 4:**
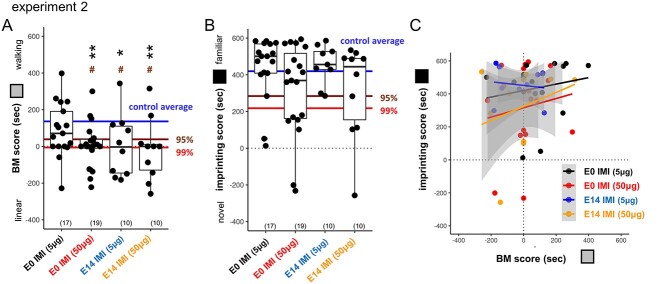
BM preference and imprinting; IMI (experiment 2). A) IMI suppressed BM if injected on E0 or E14. B, C) Although not significant, imprinting was slightly suppressed by IMI 50 μg in both injection days.

Blockade of nAChR transmission by imidacloprid (IMI) also impaired BM at low doses. When applied at E14, IMI suppressed fetal movement at the low dose (5–50 μg/egg: 0.1–1.0 ppm; see Supplementary Fig. S1B) and significantly impaired BM but spared imprinting in hatchlings (Fig. 4). As the mammalian fetus could be maternally exposed to ambient neonicotinoids, we tested the IMI effect injected into fertilized eggs before incubation (E0). The treated hatchlings showed a significant impairment of BM at 50 μg/egg. Plotting the imprinting score against the BM (Fig. 4C) revealed that the chicks with a low BM score tended to show a lower imprint score in the high-dose IMI groups (50 μg/egg), though without significant correlations (*r* = +0.24 and *P* = 0.314 for E0, *r* = +0.18 and *P* = 0.623 for E14).

### Significant effects by VPA and nAChR blockade were abolished by posthatch injection of bumetanide; shared pathology at the molecular level

Injection of bumetanide (blocker of a chloride cotransporter, NKCC1) on E14 also impaired the posthatch BM preference, whereas imprinting was spared (see Supplementary Figs. S3 and S4), suggesting a critical importance of the excitation/inhibition balance during the late phase of fetal development. On the other hand, when bumetanide was applied to P1 chicks (30 min before the start of the training session; i.v. injection of 0.02 mg/chick), statistically significant effects were abolished in both imprinting (for VPA) and BM preference (for ketamine and tubocurarine; Fig. 3A and B). Despite distinct pathogenesis, BM and imprinting impairments could share a common molecular pathology associated with GABA-AR actions. Notice also that bumetanide and VU0463271 (KCC2 blocker) had no significant effects on the spontaneous movements of E14 fetuses (Supplementary Fig. S1B). Suppression of the fetal movements is not necessary for the BM impairment.

### Imprinting of BM image was impaired by both VPA and nAChR blockade; shared behavioral phenotype

**Fig. 5:**
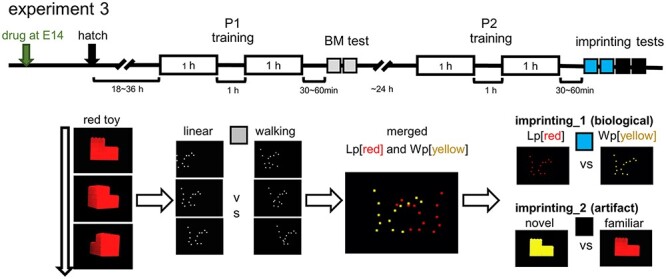
Imprinting of BM image; training and test procedures (experiment 3). After the first (P1) training using an artifact (rotating red toy) and BM test, chicks received the second day (P2) training, wherein two images of point light animations were presented with yellow in walking (Wp[yellow]) and red in linear rigid motion (Lp[red]).

BM preference canalizes subsequent imprinting (Miura and Matsushima 2016; Miura et al. 2020). If chicks were trained using a nonbiological artificial image (rotating toy in red) on P1, they oriented to a biological object (walking point in yellow, Wp[yellow]) on the subsequent day (P2; experiment 3, Fig. 5).

**Fig. 6:**
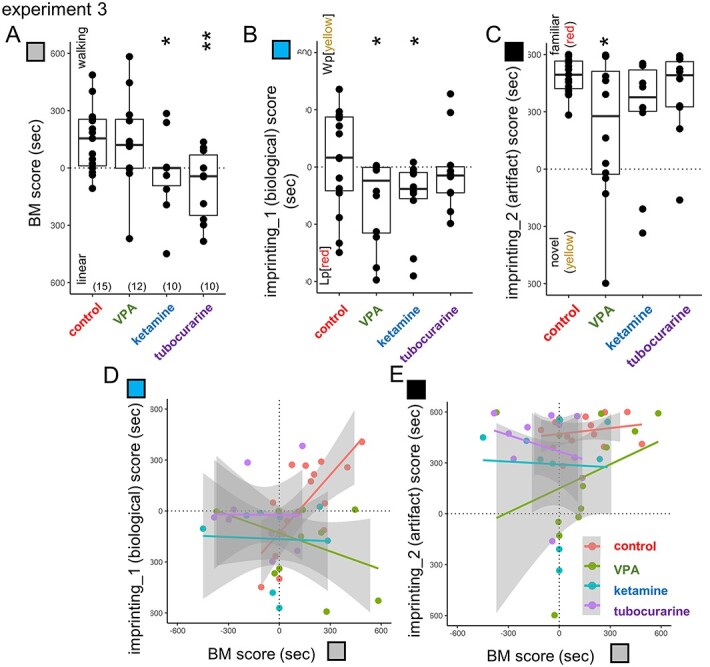
Imprinting of BM image was similarly impaired by both VPA and nAChR blockade. The 2-step imprinting paradigm revealed a common hypoplasia by E14 injection of VPA and nAChR blockade (experiment 3). A) BM scores confirmed the experiment 2. B, D) Both VPA and ketamine chicks showed the impaired formation of preference to Wp[yellow]. Notably, the tubocurarine chicks showed similar patterns of imprinting versus BM plots to the ketamine. C, E) VPA chicks showed the impaired imprinting to artifact red toy as in experiment 2.

Injection of ketamine and tubocurarine (but not VPA) on E14 suppressed BM preference on P1 (Fig. 6A) as in experiment 2. P2 training using merged point-light animations (linear motion in red and walking in yellow, Lp[red] + Wp[yellow]) impaired the preference for Wp[yellow] in all groups (imprinting_1 score in Fig. 6B). Note that preference to the artificial image (red toy) remained intact in ketamine and tubocurarine chicks, except with VPA injection (imprinting_2 score in Fig. 6C). Multiple regression analysis of the imprinting_1 score revealed a significant interaction of BM score and each of VPA (*P* = 0.000911), ketamine (*P* = 0.0165), and tubocurarine (*P* = 0.0273); those control chicks with high BM scores tended to show high imprinting_1 scores, but the VPA chicks did not. Despite distinct neuro-developmental processes, both VPA and nAChR blockade similarly impaired the attachment formation to the BM image.

### Distinct effects of VPA on histone acetylation, brain size, and neuronal maturation

**Fig. 7:**
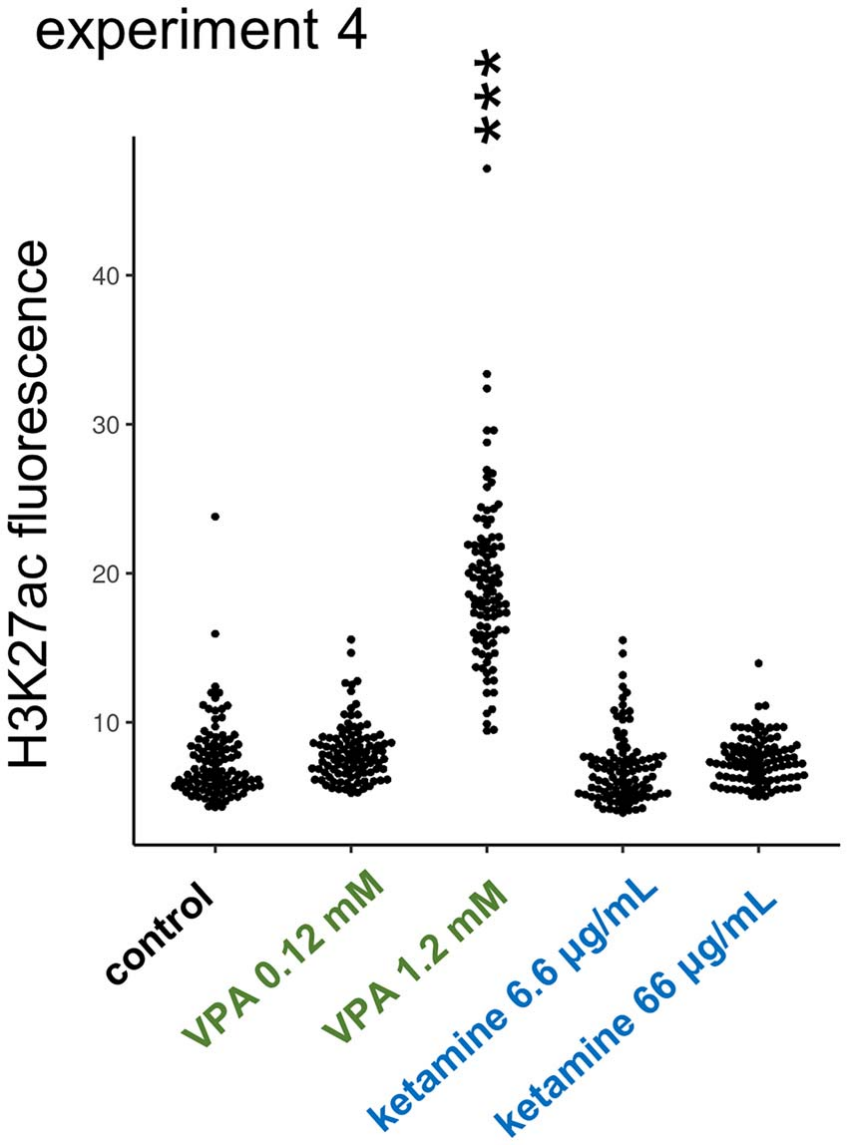
Distinct effects of VPA on histone acetylation. VPA but not ketamine enhanced the acetylation level of histone H3K27 in primary cell culture made from E14 telencephalon (experiment 4).

VPA is reported to act as a potent inhibitor of histone deacetylases (HDACs) (Phiel et al. 2001). To see if ketamine (asnAChR blockader) could also modify HDACs (Potter and Choudhury 2014; McMillan and Muthukumaraswamy 2020), we examined histone acetylation in primary cell culture prepared from E14 embryonic telencephalon; fluorescence level was measured in randomly selected 100 cells for each treatment (experiment 4, Fig. 7). As expected, VPA increased the H3K27 acetylation level at a dosage comparable to the “in ovo” injection (0.12~1.2 mM of medium), whereas ketamine had no effects at 6.6 μg/mL and 66 μg/mL, which was higher than the in ovo dose (0.2 mg/50 g = ca. 4 μg/mL). The VPA effect on HDACs was confirmed, whereas no comparable effects were found by ketamine.

**Fig. 8:**
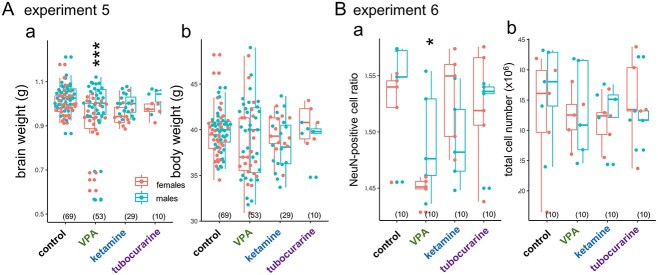
Distinct effects of VPA on brain size, and matured neuron ratio. Aa) VPA but not ketamine/tubocurarine reduced the brain weight and Ba) the ratio of NeuN positive cells measured by isotropic fractionation; Ab) body weight and Bb) total cell number were not affected. Male brains were significantly heavier, but no interaction was found between the sex and the agents. No significant effects of sex were detected on the cell number and the NeuN ratio.

Different brain morphologies could appear in these chicks with fetal chemical treatments. Although controversial, macrocephaly (increased head/brain size) is associated with some ASD subtypes due to altered neurogenesis in the early infantile period (Courchesne et al. 2003; Klein et al. 2013). In chicks, the whole-brain weight of hatchlings revealed that E14 injection of VPA, but not ketamine, significantly decreased the brain weight compared to control (experiment 5, Fig. 8Aa) without changes in the body weight (Fig. 8Ab). Notably, 11 out of 53 chicks (~21%) had a distinctly small brain, whereas the rest remained in the control range. Male brains were significantly bigger, but no significant interaction occurred between sex and treatment. Isotropic fractionation revealed a significantly lower ratio of NeuN-positive cells in VPA (Fig. 8Ba) without changes in the total cell number (Fig. 8Bb). Perturbation of gene expression by VPA could lead to the retardation of brain development during the late fetal stage and, hence, the delayed maturation of neurons.

In conclusion, suppression of fetal movement is not sufficient or necessary for the impairment of social attachments in neonates. Instead, the present study revealed 2 critical neurodevelopmental processes, namely the BM predisposition and the memory formation; the former depends on the nAChR transmission, whereas the latter is particularly fragile to VPA. Despite distinct, both processes are critical for hatchlings to form social attachments selectively to natural counterparts and share a common pathology of delayed GABA switch when impaired. Finally, IMI impaired both BM and imprinting at the sublethal dose of 1 ppm of the egg tissue.

## Discussion

### Scenario of the adaptive socialization through imprinting

**Fig. 9:**
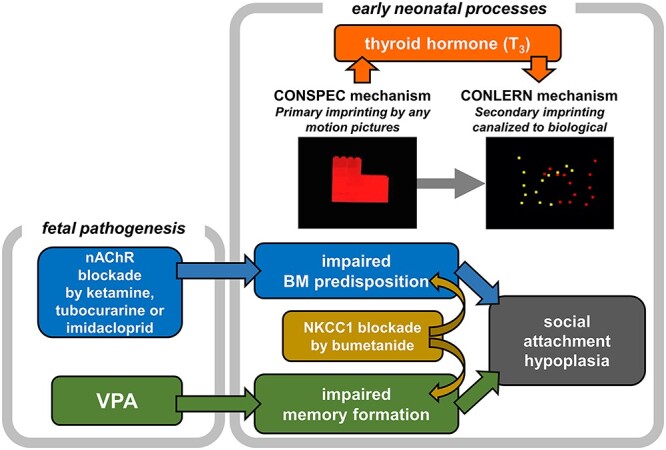
Scenario of the adaptive socialization through imprinting.

Since Lorenz (1937), imprinting has been assumed as a simple but unusual type of learning found in limited precocial animals, where irreversible 1-time learning occurs in a restricted short critical period after birth. This study series suggest a different figure: Imprinting is composed of multiple processes and mechanisms functioning together (Fig. 9). In the early neonatal processes, exposure to any moving object leads to the CONSPEC mechanism (Morton and Johnson 1991) associated with the thyroid hormone (specifically, triiodo-thyronine T_3_) influx, which is induced by the “primary imprinting” and, in turn, elongates the sensitive period (Yamaguchi et al. 2012) and enhances innate predispositions (Miura et al. 2018; Takemura et al. 2018). Therefore, subsequent “secondary imprinting” is canalized to objects bearing biological features, namely, the CONLERN mechanism is activated toward adaptive social attachment. These early processes are homologous to that proposed for neonatal development in humans (Hirai and Senju 2020).

Choice of visual stimuli warrants a careful consideration in this context. As discussed in Introduction, human neonates show a preferential looking to BM, and several factors of the visual stimuli have been suggested to underpin the preference. Hirai and Senju (2020) hypothesizes the “step detector,” which is sensitive to the characteristic configuration of “foot-below-the-body” at the bottom; importance of the local trajectory of the feet motion is stressed. Accordingly, Bardi et al. (2011) reported the newborns’ BM preference by using a nontranslational point-light animation depicting a walking hen, suggesting the importance of local cues. On the other hand, Bidet-Ildei et al. (2014) revealed that translational displacement significantly contributes; importance of the horizontal global motion of the whole point-light set is stressed. In the present study, we are unable to judge if the translational factor is critical also in chicks because subjects were tested by the binary choice between walking and linear animations, both of which contained identical horizontal displacements. Further behavioral examinations on the contributing factors will help us to figure out the neural basis of the assumed CONLERN mechanism.

### Neonicotinoids impairs neonatal visual predisposition at the dose significantly lower than the acute pathogenetic level

Environmental risk of neonicotinoids was initially related to the population decline of insectivorous birds (Hallmann et al. 2014) and the delayed migration by impaired foraging behavior (Eng et al. 2019). Recently, the association between ambient neonicotinoids and ASD in humans has been suggested in several extensive epidemiological studies (Gunier et al. 2017; von Ehrenstein et al. 2019), although the surveys are still not exhaustive and controversial particularly on the type of neonicotinoids that matter. On the other hand, the fetal/neonatal exposure of acetamiprid neonicotinoid caused abnormal development of socio-sexual behavior in mice (Sano et al. 2016). Despite the low level of acute toxicity, low affinity for vertebrate nAChR, and rapid metabolism (Bean et al. 2019), neonicotinoids are known for a high persistence level. As shown in this study, identical effective dosage appeared in the E0 and E14 groups of IMI treatment (Fig. 4). IMI suppressed BM preference nearly completely at 1 ppm (1 mg/kg egg weight), which was significantly lower than the lethal dose (LD_50_ < 44 mg/kg/day in red-legged partridges) and even lower than the dose that reduced immune response of the offspring (8.8 mg/kg/day) (Lopez-Antia et al. 2015).

### Biological and technical advantages of the chick model for screening ASD risk agents

This study suggests that the domestic chick is a bio-psychologically valid animal model (Wilner 1984; Belzung and Lemoine 2011) for studying ASD. (i) Chick has construct validity because there are common causes and processes, such as VPA, blockade of nAChR, and involvement of GABA transmission. (ii) Chick has face validity because BM preference and learned social attachment formation are assayed. (iii) Chick has predictive validity because bumetanide abolished the impairments by VPA and nAChR blockade. It must be noticed that bumetanide has been reported as a possible therapeutic agent for reducing the severity in some cases of ASD (Lemonnier et al. 2017, also see Sprengers et al. 2021). Beside the impaired social interactions, ASD is characterized by other symptoms, such as delayed speech, learning disability, repetitive stereotyped behaviors, and altered sensory perception, which we may address using chicks as the model. A battery of behavioral paradigms is already available in domestic chicks (Rugani et al. 2009; Rugani 2017; Daisley et al. 2021), ranging from numerical comprehension, arithmetic, and transitive inference associated with the social rank.

The chick model has several technical advantages. (i) Maternal complication is disregarded, whereas gestational effects are inevitably subjected to the maternal metabolism in mammals. (ii) The time course and effective dose of agents are precisely determined. (iii) The short incubation period facilitates speedy screening, as it takes only 8 days from the injection of chemicals to E14 eggs until the hatchling are tested on P1. (iv) Simple imprinting paradigm allows testing of predisposed preference and learning ability. Humans are often exposed to several ASD risk factors simultaneously. The chick model is suitable for examining interactions of multiple factors in a controlled manner.

### Limitations of the chick model for studying ASD

Chicks are inferior model in terms of the homological validity (Belzung and Lemoine 2011). Even though the neuro-cognitive processes are shared between mammals and birds (Güntürkün and Bugnyar 2016), pharmacokinetics of the risk agents most probably differ. While the mammalian fetuses/neonates could be repeatedly exposed to the agents by maternal intake and lactation, neonatal chicks are exposed through limited periods of the maternal oogenesis and the posthatch foraging. Beside the limited applicability of genetic tools in birds due to high degree of inbreeding disadvantage (Hemmings et al. 2012), longitudinal studies are missing. In particular, we must ask if impaired social behaviors occur in adults with all other physical developments kept normal.

### What is the target of the nAChR blockade?

Nicotinic transmission is critical for neurodevelopment (Liu et al. 2006; Dwyer et al. 2009), including the pathophysiology of ASD (Karvat and Kimchi 2014; Marotta et al. 2020). However, as nAChR is widespread in the brain and body (Millar and Gotti 2009; Fisher and Wonnacott 2012), several brain regions/organs must be considered. Among the possible candidates, the following regions need specific attention: (i) cholinergic neurons in the striatum and the basal forebrain projecting to wide areas in pallium (or cortex in mammals) (Ahmed et al. 2019), (ii) cholinergic neurons in nucleus isthmi parvocellularis (or pedunculo-pontine tegmental nucleus) projecting to optic tectum (superior colliculus) (Knudsen 2011), and (iii) preganglionic cholinergic terminals of parasympathetic nerve acting on thyroid gland (Iimura et al. 2019). If (i) is the case, delayed GABA switch in the pallial center for imprinting (intermediate medial mesopallium, IMM; Horn 2004) must be considered. If (ii) is the case, retarded visual attention due to the underdeveloped tecto-isthmo-tectal network must be considered. If (iii) is the case, hypothyroidism during gestation or early neonatal period must be considered as reported in human neonates (Román et al. 2013; Getahun et al. 2018) and chicks (Yamaguchi et al. 2012; Miura et al. 2018; Takemura et al. 2018). Specification of the responsible regions will enable us to design the appropriate strategy for each phenotype associated with ASD.

## Supplementary Material

nAChR_VPA_BM_supplementary_materials_tgac041
