# Supplementary material for: Fetal blockade of nicotinic acetylcholine transmission causes autism-like impairment of biological motion preference in the neonatal chick
Source: Cereb Cortex Commun. 2022 Nov 18;3(4):tgac041. doi: 10.1093/texcom/tgac041 (PMC10478028; doi:10.1093/texcom/tgac041)
Supplement: nAChR_VPA_BM_supplementary_materials_tgac041 [file nachr_vpa_bm_supplementary_materials_tgac041.docx]

**Supplementary materials**

Fetal Blockade of Nicotinic Acetylcholine Transmission Causes Autism-like Impairment of Biological Motion Preference in the Neonatal Chick

Matsushima et al. (2022) *Cerebral Cortex Communications*

<https://doi.org/10.1093/texcom/tgac041>

Corresponding author:

Toshiya Matsushima (Hokkaido University)

Orcid ID: 0000-0001-9404-647X

Email address: [matsushimatoshiya@gmail.com](mailto:matsushimatoshiya@gmail.com)

The dataset (raw data in excel format and the RStudio codes in html files) and the point light animations used in this study (wmv files) are available at the repository site of the Hokkaido University (HUSCAP):

<http://hdl.handle.net/2115/87070>

Preprint: is available at bioRxiv; <https://www.biorxiv.org/content/10.1101/2022.05.19.492744v4>

prepared on November 7, 2022, by Toshiya Matsushima

**Contents**

- Coding of chicks for blinded behavioral tests
- List of the point-light animations for training and tests
- List of the chemical agents
- Ballistographic recording of fetal movements (experiment 1)
- Determination of sample size based on a plot experiment
- Statistical computation
  - Experiment 1 Ballistography index explained by drug (Figure 1C)
  - Experiment 2, VPA, ketamine, mk801, tubocurarine, MLA and DHβE (Figure 3)
    - BM score explained by drug (Figure 3a)
    - imprinting score explained by drug (Figure 3b)
  - Experiment 2, imidacloprid (Figure 4)
    - BM score explained by drug (Figure 4a)
    - imprinting score explained by drug (Figure 4b)
  - BM and imprinting scores of all of the experimental groups examined in this study
  - Bootstrapping analysis of the Experiment 2 data
  - Experiment 3 (Figure 6)
    - BM score explained by drug (Figure 6a)
    - imprinting_1 (biological) score explained by drug (Figure 6b)
    - imprinting_1 (biological) score explained by drug and BM score (Figure 6b)
    - imprinting_2 (artifact) score explained by drug (Figure 6c)
    - imprinting_2 (artifact) score explained by drug and BM score (Figure 6c)
  - Experiment 4 (Figure 7) H3K27ac fluorescence explained by drug
  - Experiment 5 and 6 (Figure 8A, B)
    - brain weight (Figure 8Aa)
    - body weight (Figure 8Ab)
    - NeuN-positive cell ratio (Figure 8Ba)
    - total cell number (Figure 8Bb)

**Coding of chicks for blinded behavioral tests**

On embryonic day 14 (E14), eggs of the same batch were randomly assigned to experimental groups of different chemicals/doses and control group, which received a vehicle injection (200μL of distilled water) to the air sac. The eggs were coded by the date of injection (e.g., 220101 for 1 January 2022), the ordinal numbers (starting from 1, according to the order of injection), chemical name and the dose, such as “220101-1-vpa-35” for an egg injected 35μmole VPA on 1 January of 2022. After hatch, chicks were re-coded by the day and the hatching order, such as “220108-1” for a chick that hatched first on 8 January 2022 (for the chicks handled by Matsushima T). For another set of chicks handled by Miura M, chicks were given separate series of id number such as “id100”, again without specification of the chemical treatment.

**List of the point-light animations for training and tests**

| Moving-toy(red).wmv | P1 training and imprinting test (experiment 2,3) |
| --- | --- |
| Moving-toy(yellow).wmv | imprinting test (experiment 2,3) |
| Wp(white).wmv | BM test (experiment 2,3) |
| Lp(white).wmv | BM test (experiment 2,3) |
| Wp(yellow)-Lp(red).wmv | P2 training (experiment 3) |
| Wp(yellow).wmv | imprinting_1 (biological) test (experiment 3) |
| Lp(red).wmv | imprinting_1 (biological) test (experiment 3) |

**List of the chemical agents**

*Name, code and company of the chemical agents*

| chemical name | code number | company |
| --- | --- | --- |
| VPA (sodium valproate) | cas no.1069-66-5 | FUJIFILM Wako Pure Chemical Co. |
| ketamine (hydrochloride) | ATC code N01AX03 | Daiichi-Sankyo Propharma Co. |
| mk801 ((+)-mk801 maleate) | cas no. 77086-22-7 | FUJIFILM Wako Pure Chemical Co. |
| tubocurarine (D-tubocurarine chloride pentahydrate) | cas no. 41354-45-4 | FUJIFILM Wako Pure Chemical Co. |
| MLA (methyllycaconitine citrate) | cas no. 112825-05-5 | abcam |
| DHβE (dihydro-β-erythroidine hydrobromide) | cas no. 29734-68-7 | TOCRIS Bioscience |
| imidacloprid (standard) | cas no. 105827-78-9 | FUJIFILM Wako Pure Chemical Co. |
| bumetanide | cas no. 28395-03-1 | TOCRIS Bio-techne |
| VU0463271 | cas no. 1391737-01-1 | TOCRIS Bioscience |

*Amount of agents injected / egg ~ 50g*

| chemical agent | molecular weight | μmole |
| --- | --- | --- |
| VPA 5.82mg | 166.19 | 35.0 |
| ketamine 0.20mg | 274.19 | 0.729 |
| mk801 0.25mg | 337.37 | 0.741 |
| tubocurarine 0.20mg | 771.72 | 0.259 |
| MLA 0.15mg | 874.92 | 0.171 |
| DHβE 0.15mg | 356.26 | 0.421 |
| imidacloprid 0.05mg | 255.66 | 0.196 |
| bumetanide 0.02mg | 364.42 | 0.0549 |
| VU0463271 0.2mg | 387 | 0.517 |

**Ballistographic recording of fetal movements (experiment 1)**

**
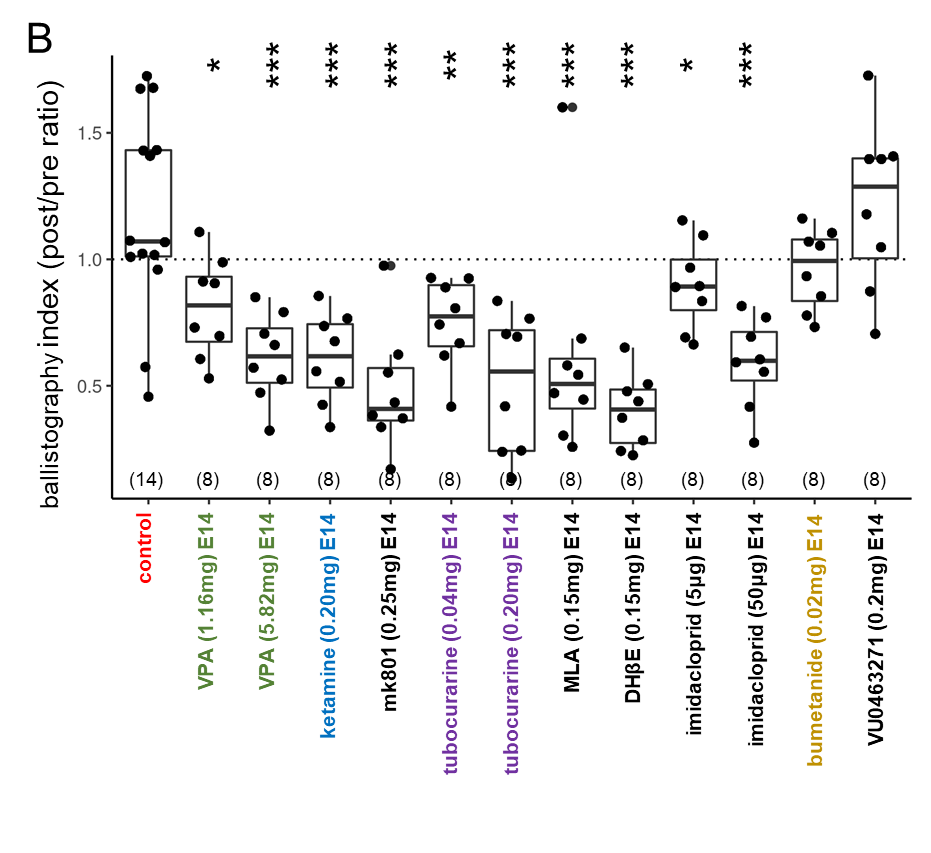

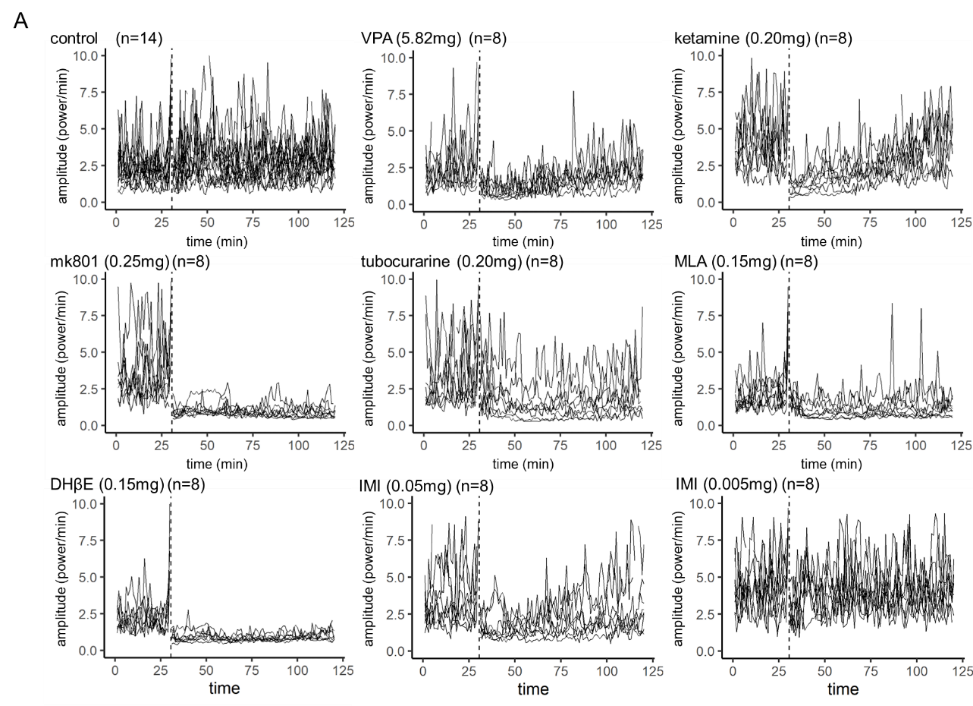
**Superimposed traces of the ballistography obtained from 9 groups of fetuses are shown below (**Figure S1A**). In addition, dose dependency of the suppressive effects of VPA, tubocurarine and IMI was examined (**Figure S1B**). Sample size was arbitrarily set as n=8 for each group of chemical/dosage, and the present study is based on recordings obtained from 110 eggs in total.

**Figure S1A and B**

**Determination of sample size based on a plot experiment**

Sample size and stimulus used for training (video clips) could not be determined *a pri ori*. Instead, we accomplished a pilot experiment using two groups of chicks (n=10 and 11) as shown below (**Figure S2**); one group was trained by red toy (**Aa**) and another by yellow (**Ab**).


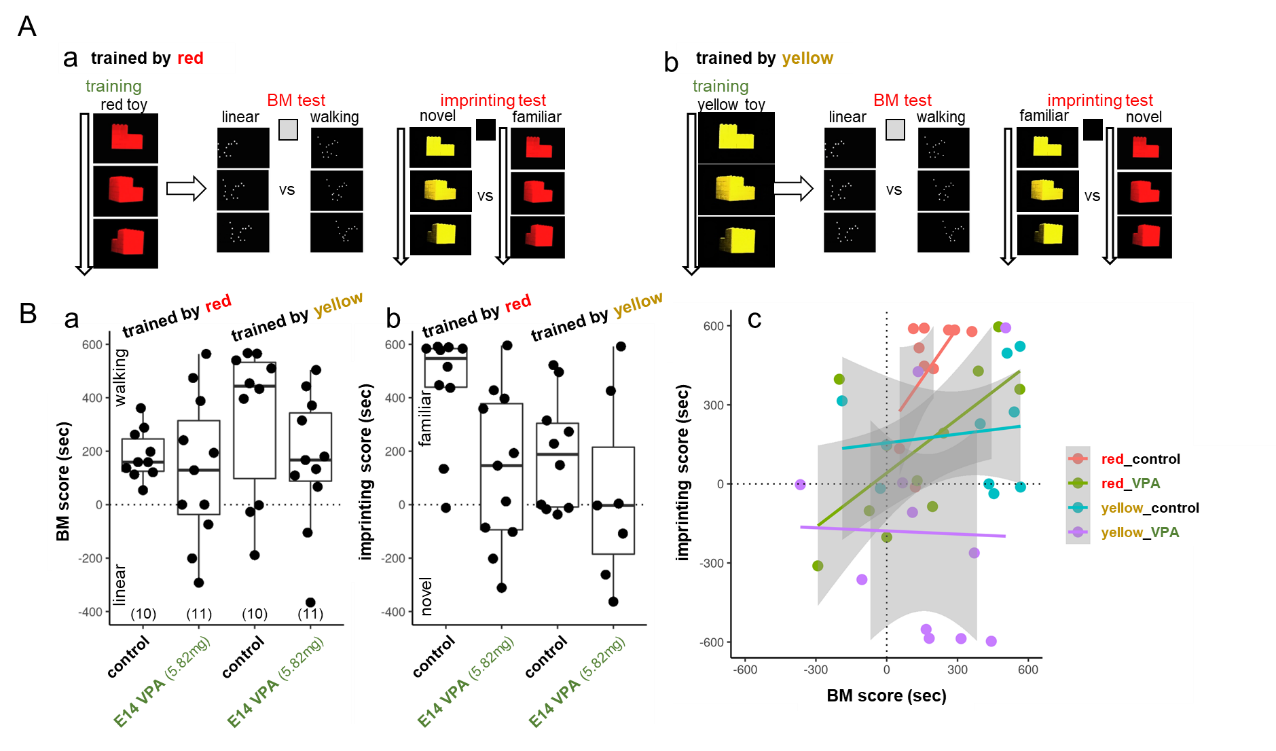
**Figure S2**

Summary statistics of the behavioral scores are the following:

- BM scores:

|  | n | mean | standard deviation |
| --- | --- | --- | --- |
| red_control | 10 | 185.2 | 92.963 |
| red_VPA | 11 | 129.364 | 274.359 |
| yellow_control | 10 | 324.700 | 283.821 |
| yellow_VPA | 11 | 165.273 | 250.199 |

- imprinting scores:

|  | n | mean | standard deviation |
| --- | --- | --- | --- |
| red_control | 10 | 444.800 | 213.193 |
| red_VPA | 11 | 130. | 292.066 |
| yellow_control | 10 | 191.600 | 211.039 |
| yellow_VPA | 11 | -185.091 | 414.279 |

Though chicks consistently showed a biased preference to red, ANOVA revealed significantly different imprinting scores between VPA and control without interaction with the color. No significant differences appeared in the bm scores.

- BM scores:


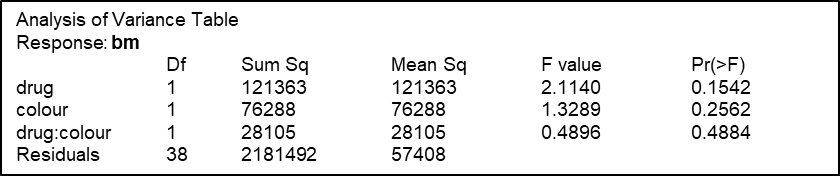


- imprinting scores:


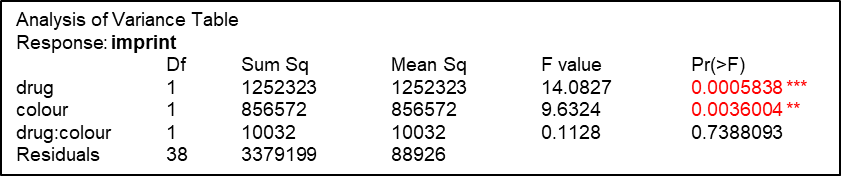


The effect size (Cohen’s d value) was computed for each pairwise comparison between VPA and control such as:

|  | trained by red | trained by yellow |
| --- | --- | --- |
| BM scores | 0.2675 | 0.5976 |
| imprint scores | 1.223 | 1.130 |

As considerable effect sizes were obtained for imprinting scores (both higher than 1.0), we concluded that the sample size =10 yields reliable statistical judgements. We also decided to train chicks using red toy, because no significant interactions were found, despite clear color preference to red over yellow. It is also notable that VPA did not impair the predisposed red color preference (see Miura et al. 2020).

In systematically examining the effects of chemicals, we adopted two levels of dose, namely high dose following the ballistography experiment, and low dose set 1/5 of the high dose. For each batch of eggs, 1 control group and 2 experimental groups were prepared. When a pre-determined sample size (n=10) was accumulated for each chemical/dose, behavioral experiments were not replicated. In total, 23 experimental groups (n=10) and one control (n=123) were thus collected (see below **Figure S4**). The control data somehow fluctuated in different batches, but no systematic batch-dependency was found and thus merged. As noted above in ***1.3.***, to examine the effects of IMI injected to E0 eggs, we obtained more than 10 chicks (17 for 5μg, 19 for 50μg). In addition, we had 11 chicks in the ketamine (E14) + bumetanide (P1) experiment by mistake. The experiment 2 was therefore based on a total of 370 chicks.

**Statistical computation**

**Experiment 1**

**Ballistography index explained by drug (Fig. 1C)**

*fit_exp_1_ballistography_selected <- lm(ratio ~ label, data = dataset_exp_1_ballistography_fig)*

*summary(fit_exp_1_ballistography_selected)*

Coefficients:

| label (drug) | estimate | std. error | t-value | Pr(>\|t\|) | signif. code |
| --- | --- | --- | --- | --- | --- |
| (intercept) | 1.18063 | 0.07899 | 14.947 | < 2e-16 | *** |
| VPA 5.82mg | -0.56805 | 0.13099 | -4.337 | 6.23e-05 | *** |
| ketamine 0.20mg | -0.57210 | 0.13099 | -4.368 | 5.60e-05 | *** |
| mk801 0.25mg | -0.69986 | 0.13099 | -5.343 | 1.80e-06 | *** |
| tubocurarine 0.20mg | -0.67630 | 0.13099 | -5.163 | 3.46e-06 | *** |
| MLA 0.15mg | -0.56939 | 0.13099 | -4.347 | 6.01e-05 | *** |
| DHβE 0.15mg | -0.78066 | 0.13099 | -5.960 | 1.87e-07 | *** |

residual standard error = 0.2956 (Df = 55)

multiple r-squared = 0.5006, adjusted r-squared = 0.4461

F-statistic = 9.188 (Df = 6, 55), p-value = 5.765e-07

**Experiment 2, VPA, ketamine, mk801, tubocurarine, MLA and DHβE (Fig 3)**

**BM score explained by drug (Fig. 3A)**

*fit_exp2_bm_selected <- lm (bm ~ label, data = dataset_exp_2_bm_imprint_fig)*

*summary (fit_exp2_bm_selected)*

Coefficients:

| drug | estimate | std. error | t-value | Pr(>\|t\|) | signif. code |
| --- | --- | --- | --- | --- | --- |
| (intercept) | 136.309 | 16.187 | 8.415 | 6.86e-15 | *** |
| VPA 5.82mg | 26.091 | 59.075 | 0.442 | 0.65920 | ns |
| VPA 5.82mg +bumetanide | -53.709 | 59.075 | -0.909 | 0.36434 | ns |
| ketamine 0.20mg | -146.809 | 59.075 | -2.485 | 0.01375 | * |
| ketamine 0.20mg  +bumetanide | -92.854 | 56.537 | -1.642 | 0.10206 | ns |
| mk801 0.25mg | -9.509 | 59.075 | -0.161 | 0.87228 | ns |
| tubocurarine 0.20mg | -162.509 | 59.075 | -2.751 | 0.00648 | ** |
| tubocurarine 0.20mg +bumetanide | -71.109 | 59.075 | -1.204 | 0.23010 | ns |
| MLA 0.15mg | -153.509 | 59.075 | -2.599 | 0.01005 | * |
| DHβE 0.15mg | -50.309 | 59.075 | -0.852 | 0.39543 | ns |

residual standard error = 179.7 (Df=204)

multiple r-squared = 0.09335, adjusted r-squared = 0.05335

F-statistic = 2.334 (Df = 9, 204), p-value = 0.01597

**imprinting score explained by drug (Fig. 3B)**

*fit_exp2_imprint_selected <- lm (imprint ~ label, data = dataset_exp_2_bm_imprint_fig)*

*summary (fit_exp2_imprint_selected)*

Coefficients:

| drug | estimate | std. error | t-value | Pr(>\|t\|) | signif. code |
| --- | --- | --- | --- | --- | --- |
| (intercept) | 419.52 | 19.80 | 21.183 | < 2e-16 | *** |
| VPA 5.82mg | -316.22 | 72.22 | -4.378 | 1.91e-05 | *** |
| VPA 5.82mg +bumetanide | 84.68 | 72.22 | 1.172 | 0.2424 | ns |
| ketamine 0.20mg | -118.62 | 72.22 | -1.642 | 0.1020 | ns |
| ketamine 0.20mg  +bumetanide | -26.07 | 69.12 | -0.377 | 0.7065 | ns |
| mk801 0.25mg | 3.88 | 72.22 | 0.054 | 0.9572 | ns |
| tubocurarine 0.20mg | 47.08 | 72.22 | 0.652 | 0.5152 | ns |
| tubocurarine 0.20mg +bumetanide | -5.42 | 72.22 | -0.075 | 0.9402 | ns |
| MLA 0.15mg | -160.32 | 72.22 | -2.220 | 0.0275 | * |
| DHβE 0.15mg | -151.02 | 72.22 | -2.091 | 0.0378 | * |

residual standard error = 219.6 (DF = 204)

multiple r-squared = 0.1348, adjusted r-squared = 0.09664

F-statistic = 3.532 (DF = 9, 204), p-value = 0.0004322

**Experiment 2, imidacloprid (Fig. 4)**

**BM score explained by drug (Fig. 4A)**

*fit_exp_3_bm <- lm (bm ~ label, data = dataset_exp_2_bm_imprint_ctrl_imi)*

*summary (fit_exp_3_bm)*

Coefficients:

| drug | estimate | std. error | t-value | Pr(>\|t\|) | signif. code |
| --- | --- | --- | --- | --- | --- |
| (intercept) | 136.31 | 15.34 | 8.884 | 7.85e-16 | *** |
| E0 IMI 0.005mg | - 46.90 | 44.03 | - 1.065 | 0.28829 | ns |
| E0 IMI 0.05mg | - 128.62 | 41.94 | - 3.067 | 0.00251 | ** |
| E14 IMI 0.005mg | -114.16 | 55.95 | - 2.374 | 0.01871 | * |
| E14 IMI 0.05mg | - 155.71 | 55.95 | - 2.783 | 0.00598 | ** |

residual standard error = 170.2 (DF = 174)

multiple r-squared = 0.1002, adjusted r-squared = 0.0795

F-statistic = 4.843 (DF = 4, 174), p-value = 0.0009977

**imprinting score explained by drug (Fig. 4B)**

*fit_exp_3_imprint <- lm (imprint ~ label, data = dataset_exp_2_bm_imprint_ctrl_imi)*

*summary (fit_exp_3_imprint)*

Coefficients:

| drug | estimate | std. error | t-value | Pr(>\|t\|) | signif. code |
| --- | --- | --- | --- | --- | --- |
| (intercept) | 419.52 | 20.85 | 20.117 | <2e-16 | *** |
| E0 IMI (0.005mg) | 18.24 | 59.84 | 0.305 | 0.7608 | ns |
| E0 IMI (0.05mg) | - 101.52 | 57.01 | - 1.781 | 0.0767 | ns |
| E14 IMI (0.005mg) | - 62.62 | 76.05 | - 0.823 | 0.4114 | ns |
| E14 IMI (0.05mg) | - 03.62 | 76.05 | - 1.363 | 0.1748 | ns |

residual standard error = 231.3 (DF = 174)

multiple r-squared = 0.03001, adjusted r-squared = 0.007709

F-statistic = 1.346 (DF = 4, 174), p-value = 0.2549

**BM and imprinting scores of all of the experimental groups examined in this study**

**
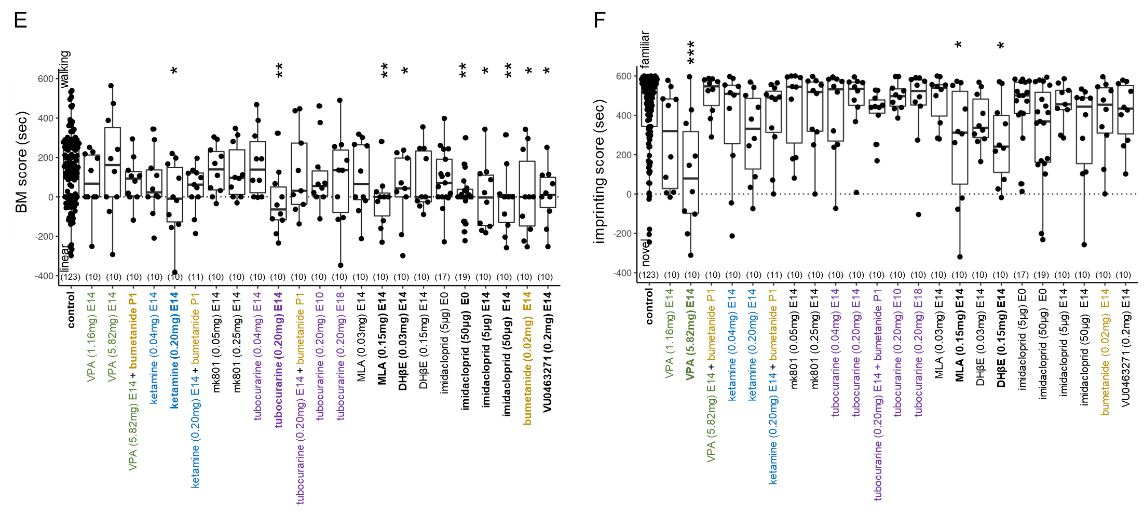
**

**Figure S3**

**Bootstrapping analysis of the Experiment 2 data**

**
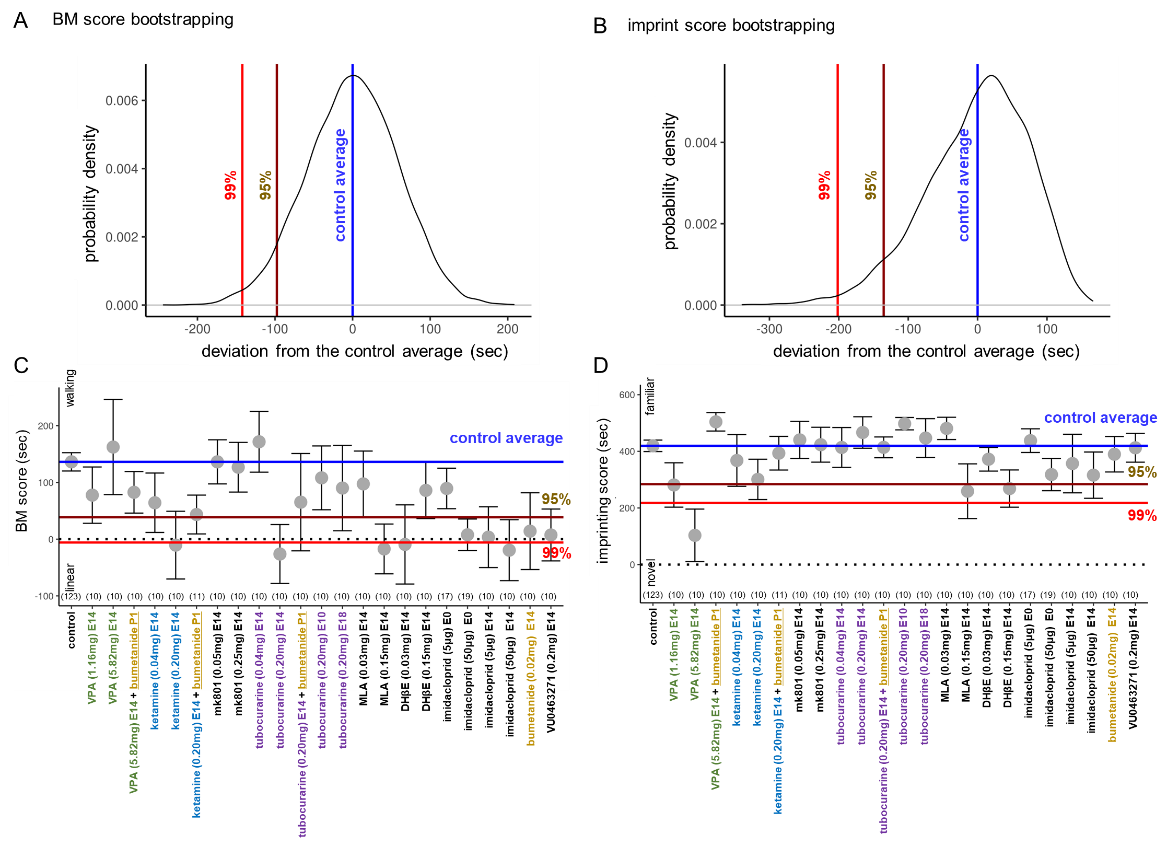
**We made post-hoc bootstrapping analysis of the control data (123 chicks) to obtain the distribution (probability density) of the mean of samples composed of n=10 chicks. For each of BM and imprinting scores, 10 chicks were randomly sampled 10,000 times from the control data. For each sample from 10 chicks, difference between the sample average (n=10) and the average of the unsampled chicks (n=113) were calculated, thus yielding a set of 10,000 values. Distribution of the difference value was represented as probability density curves shown below (**Figure S4**), and the critical points where the area below the curve occupied 95% and 99% were obtained by using *quantile()*.

**Figure S4**

**Experiment 3 (Fig. 6)**

**BM score explained by drug (Fig. 6A)**

*fit_exp3_bm_drug <- lm(bm ~ drug, data = dataset_exp_3_double_imprint)*

*summary (fit_exp3_bm_drug)*

Coefficients:

| drug | estimate | std. error | t-value | Pr(>\|t\|) | signif. code |
| --- | --- | --- | --- | --- | --- |
| control | 145.80 | 52.24 | 2.791 | 0.00781 | ** |
| VPA 5.82mg | -18.22 | 78.37 | -0.232 | 0.81729 | ns |
| ketamine 0.20mg | -172.30 | 82.61 | -2.086 | 0.04296 | * |
| tubocurarine 0.20mg | -235.00 | 82.61 | -2.845 | 0.00678 | ** |

residual standard error = 202.3 (DF = 43)

multiple r-squared = 0.2076, adjusted r-squared = 0.1523

F-statistic =3.755 (DF = 3, 43), p-value = 0.01758

**imprinting_1 (biological) score explained by drug (Fig. 6B)**

*fit_exp3_imprint_1 <- lm(imprint_1 ~ drug, data = dataset_exp_3_double_imprint)*

*summary (fit_exp3_imprint_1)*

Coefficients:

| drug | estimate | std. error | t-value | Pr(>\|t\|) | signif. code |
| --- | --- | --- | --- | --- | --- |
| control | 37.13 | 59.77 | 0.621 | 0.5377 | ns |
| VPA 5.82mg | -213.05 | 89.66 | -2.376 | 0.0220 | * |
| ketamine 0.20mg | -201.53 | 94.51 | -2.132 | 0.0387 | * |
| tubocurarine 0.20mg | -60.33 | 94.51 | -0.638 | 0.5266 | ns |

residual standard error = 231.5 (DF = 43)

multiple r-squared = 0.1533, adjusted r-squared = 0.09428

F-statistic = 2.596 (DF = 3, 43), p-value = 0.06465

**imprinting_1 (biological) score explained by drug and BM score (Fig. 6B)**

*fit_exp3_imprint_1_bm <- lm(imprint_1 ~ drug * bm, data = dataset_exp_3_double_imprint)*

*summary (fit_exp3_imprint_1_bm)*

Coefficients:

| drug/BM | estimate | std. error | t-value | Pr(>\|t\|) | signif. code |
| --- | --- | --- | --- | --- | --- |
| (intercept) | -128.6864 | 72.0850 | -1.785 | 0.082007 | ns |
| VPA 5.82mg | -1.7390 | 99.4170 | -0.017 | 0.986134 | ns |
| ketamine 0.20mg | -36.8073 | 98.1233 | -0.375 | 0.709609 | ns |
| tubocurarine 0.20mg | 104.6133 | 103.3479 | 1.012 | 0.317660 | ns |
| BM | 1.1373 | 0.3286 | 3.462 | 0.001317 | ** |
| VPA*BM | -1.4939 | 0.4161 | -3.591 | 0.000911 | *** |
| ketamine*BM | -1.1786 | 0.4706 | -2.505 | 0.016545 | * |
| tubocurarine*BM | -1.1471 | 0.5003 | -2.293 | 0.027323 | * |

residual standard error = 208.6 (DF = 39)

multiple r-squared = 0.3764, adjusted r-squared = 0.2645

F-statistic = 3.363 (DF = 7, 39), p-value = 0.00668

**imprinting_2 (artifact) score explained by drug (Fig. 6C)**

*fit_exp3_imprint_2 <- lm(imprint_2 ~ drug, data = dataset_exp_3_double_imprint)*

*summary (fit_exp3_imprint_2)*

Coefficients:

| drug | estimate | std. error | t-value | Pr(>\|t\|) | signif. code |
| --- | --- | --- | --- | --- | --- |
| control | 487.13 | 68.66 | 7.095 | 9.38e-09 | *** |
| VPA 5.82mg | -277.38 | 102.99 | -2.693 | 0.0100 | * |
| ketamine 0.20mg | -194.93 | 108.56 | -1.796 | 0.0796 | ns |
| tubocurarine 0.20mg | -91.23 | 108.56 | -0.840 | 0.4053 | ns |

residual standard error = 265.9 (DF = 43)

multiple r-squared = 0.1582, adjusted r-squared = 0.09942

F-statistic = 2.693 (DF = 3, 43), p-value = 0.0579

**imprinting_2 (artifact) score explained by drug and BM score (Fig. 6C)**

*fit_exp3_imprint_2_bm <- lm(imprint_2 ~ drug*bm, data = dataset_exp_3_double_imprint)*

*summary (fit_exp3_imprint_2_bm)*

Coefficients:

| drug/BM | estimate | std. error | t-value | Pr(>\|t\|) | signif. code |
| --- | --- | --- | --- | --- | --- |
| (intercept) | 470.3160 | 93.4620 | 5.032 | 1.13e-05 | *** |
| VPA 5.82mg | -320.8333 | 128.8993 | -2.489 | 0.0172 | * |
| ketamine 0.20mg | -179.5913 | 127.2220 | -1.412 | 0.1660 | ns |
| tubocurarine 0.20mg | -103.0923 | 133.9960 | -0.769 | 0.4463 | ns |
| BM | 0.1153 | 0.4260 | 0.271 | 0.7880 | ns |
| VPA*BM | 0.3570 | 0.5394 | 0.662 | 0.5120 | ns |
| ketamine*BM | -0.1710 | 0.6101 | -0.280 | 0.7807 | ns |
| tubocurarine*BM | -0.4368 | 0.6486 | -0.673 | 0.5046 | ns |

residual standard error = 270.5 (DF = 39)

multiple r-squared = 0.21, adjusted r-squared = 0.06819

F-statistic = 1.481 (DF = 7, 39), p-value = 0.2026

**Experiment 4 (Fig. 7)**

**H3K27ac fluorescence explained by drug**

*fit_exp_4_h3k27ac <- lm(flu ~ drug, data = dataset_exp_4_h3k27ac)*

*summary(fit_exp_4_h3k27ac)*

Coefficients:

| drug | estimate | std. error | t-value | Pr(>\|t\|) | signif. code |
| --- | --- | --- | --- | --- | --- |
| (intercept) | 7.4293 | 0.3156 | 23.537 | <2e-16 | *** |
| VPA 0.12mM | 0.5917 | 0.4464 | 1.326 | 0.186 | ns |
| VPA 1.2mM | 11.9921 | 0.4464 | 26.865 | <2e-16 | *** |
| ketamine 6.6 μg/mL | -0.4920 | 0.4464 | -1.102 | 0.271 | ns |
| ketamine 66 μg/mL | 0.0230 | 0.4464 | 0.052 | 0.959 | ns |

residual standard error = 3.156 (DF = 495)

multiple r-squared = 0.7, adjusted r-squared = 0.6975

F-statistic = 288.7 (DF=4, 495), p-value: < 2.2e-16

**Experiment 5 and 6 (Fig. 8A and B)**

**brain weight (Fig. 8Aa)**

*fit_exp_5_abs_w_brain <- lm (w_brain ~ sex * drug, data = dataset_exp_5_brain_weight)*

*summary (fit_exp_5_abs_w_brain)*

Coefficients:

| drug/sex | estimate | std. error | t-value | Pr(>\|t\|) | signif. code |
| --- | --- | --- | --- | --- | --- |
| (intercept) | 1.00174 | 0.01520 | 65.887 | < 2e-16 | *** |
| sex | 0.03101 | 0.02135 | 1.453 | 0.148404 | ns |
| VPA 5.82mg | -0.08410 | 0.02426 | -3.467 | 0.000683 | *** |
| ketamine 0.20mg | -0.04889 | 0.02891 | -1.691 | 0.092845 | ns |
| tubocurarine 0.20mg | -0.03094 | 0.04246 | -0.729 | 0.467397 | ns |
| sex* VPA | 0.02942 | 0.03266 | 0.901 | 0.369059 | ns |
| sex* ketamine | 0.01340 | 0.03939 | 0.340 | 0.734245 | ns |
| sex* tubocurarine | 0.02419 | 0.06000 | 0.403 | 0.687336 | ns |

residual standard error = 0.08865 (DF = 153)

multiple r-squared = 0.1514, adjusted r-squared = 0.1126

F-statistic= 3.901 (Df = 7, 153), p-value = 0.0006117

**body weight (Fig. 8Ab)**

*fit_exp_5_abs_w_body <- lm (w_body ~ sex * drug, data = dataset_exp_5_brain_weight)*

*summary (fit_exp_5_abs_w_body)*

Coefficients:

| drug/sex | estimate | std. error | t-value | Pr(>\|t\|) | signif. code |
| --- | --- | --- | --- | --- | --- |
| (intercept) | 39.65882 | 0.54955 | 72.166 | <2e-16 | *** |
| sex | 0.17832 | 0.77161 | 0.231 | 0.818 | ns |
| VPA 5.82mg | -1.3451 | 0.87678 | -1.534 | 0.127 | ns |
| ketamine 0.20mg | -0.06652 | 1.04492 | -0.064 | 0.949 | ns |
| tubocurarine 0.20mg | 1.10118 | 1.53481 | 0.717 | 0.474 | ns |
| sex* VPA | 0.85643 | 1.18040 | 0.726 | 0.469 | ns |
| sex* ketamine | -1.36438 | 1.42373 | -0.958 | 0.339 | ns |
| sex* tubocurarine | -1.91832 | 2.16856 | -0.885 | 0.378 | ns |

Residual standard error = 3.204 (DF = 153)

multiple r-squared = 0.03717, adjusted r-squared = -0.006877

F-statistic= 0.8439 (Df = 7, 153), p-value = 0.5527

**NeuN-positive cell ratio (Fig. 8Ba)**

*fit_exp_6_neuron_glia_ratio <- lm(neuron_ratio ~ sex * drug, data = dataset_exp_6_neuron_glia)*

*summary (fit_exp_6_neuron_glia_ratio)*

Coefficients:

| drug/sex | estimate | std. error | t-value | Pr(>\|t\|) | signif. code |
| --- | --- | --- | --- | --- | --- |
| (intercept) | 0.522874 | 0.019104 | 27.370 | <2e-16 | *** |
| sex | 0.017086 | 0.027017 | 0.632 | 0.5316 | ns |
| VPA 5.82mg | -0.073903 | 0.027017 | -2.735 | 0.0101 | * |
| ketamine 0.20mg | 0.008096 | 0.027017 | 0.300 | 0.7664 | ns |
| tubocurarine 0.20mg | -0.001942 | 0.027017 | -0.072 | 0.9431 | ns |
| sex* VPA | 0.025310 | 0.038208 | 0.662 | 0.5124 | ns |
| sex* ketamine | -0.055518 | 0.038208 | -1.453 | 0.1559 | ns |
| sex* tubocurarine | -0.017455 | 0.038208 | -0.457 | 0.6509 | ns |

residual standard error=0.04272 (DF = 32)

multiple r-squared = 0.3429, adjusted r-squared = 0.1991

F-statistic: 2.385 (Df=7, 32), p-value = 0.04411

**total cell number (Fig. 8Bb)**

*fit_exp_6_cell_count <- lm(cell_number ~ sex * drug, data = dataset_exp_6_neuron_glia)*

*summary (fit_exp_6_cell_count)*

Coefficients:

| drug/sex | estimate | std. error | t-value | Pr(>\|t\|) | signif. code |
| --- | --- | --- | --- | --- | --- |
| (intercept) | 32.806 | 3.128 | 10.488 | 6.97e-12 | *** |
| sex | 3.626 | 4.424 | 0.820 | 0.418 | ns |
| VPA 5.82mg | -0.599 | 4.424 | -0.135 | 0.893 | ns |
| ketamine 0.20mg | -1.412 | 4.424 | -0.319 | 0.752 | ns |
| tubocurarine 0.20mg | 2.088 | 4.424 | 0.472 | 0.640 | ns |
| sex* VPA | -2.719 | 6.256 | -0.435 | 0.667 | ns |
| sex* ketamine | -2.004 | 6.256 | -0.320 | 0.751 | ns |
| sex* tubocurarine | -5.436 | 6.256 | -0.869 | 0.391 | ns |

residual standard error=6.994 (DF = 32)

multiple r-squared = 0.05307, adjusted r-squared = -0.1541

F-statistic= 0.2562 (Df = 7, 32), p-value = 0.9663
